# Full-Length Transcriptomes Reconstruction Reveals Intraspecific Diversity in Hairy Vetch (*Vicia villosa* Roth) and Smooth Vetch (*V. villosa* Roth var. *glabrescens*)

**DOI:** 10.3390/plants13233291

**Published:** 2024-11-22

**Authors:** Weiyi Kong, Bohao Geng, Wenhui Yan, Jun Xia, Wenkai Xu, Na Zhao, Zhenfei Guo

**Affiliations:** 1 College of Grassland Science, Nanjing Agricultural University, Nanjing 210095, China; kongweiyi@njau.edu.cn (W.K.); t2023007@njau.edu.cn (B.G.); 2022120019@stu.njau.edu.cn (W.Y.); 2019820019@njau.edu.cn (J.X.); 2020820022@stu.njau.edu.cn (W.X.); nazhao@njau.edu.cn (N.Z.); 2 The National Forestry and Grassland Administration Engineering Research Center for Germplasm Innovation and Utilization of Warm-Season Turfgrasses, Jiangsu Key Laboratory for the Research and Utilization of Plant Resources, Institute of Botany, Jiangsu Province and Chinese Academy of Sciences (Nanjing Botanical Garden Mem. Sun Yat-Sen), Nanjing 210014, China

**Keywords:** hairy vetch, full-length transcriptome, alternative splicing, LncRNA, positive selection

## Abstract

Hairy vetch (*Vicia villosa* Roth) and smooth vetch (*V. villosa* Roth var. *glabrescens*) are important cover crops and legume forage with great economic and ecological values. Due to the large and highly heterozygous genome, full-length transcriptome reconstruction is a cost-effective route to mining their genetic resources. In this study, a hybrid sequencing approach combining SMRT and NGS technologies was applied. The results showed that 28,747 and 40,600 high-quality non-redundant transcripts with an average length of 1808 bp and 1768 bp were generated from hairy vetch and smooth vetch, including 24,864 and 35,035 open reading frames (ORFs), respectively. More than 96% of transcripts were annotated to the public databases, and around 25% of isoforms underwent alternative splicing (AS) events. In addition, 987 and 1587 high-confidence lncRNAs were identified in two vetches. Interestingly, smooth vetch contains more specific transcripts and orthologous clusters than hairy vetch, revealing intraspecific transcript diversity. The phylogeny revealed that they were clustered together and closely related to the genus *Pisum*. Furthermore, the estimation of Ka/Ks ratios showed that purifying selection was the predominant force. A putative 3-dehydroquinate dehydratase/shikimate dehydrogenase (DHD/SDH) gene underwent strong positive selection and might regulate phenotypic differences between hairy vetch and smooth vetch. Overall, our study provides a vital characterization of two full-length transcriptomes in *Vicia villosa*, which will be valuable for their molecular research and breeding.

## 1. Introduction

Hairy vetch (*Vicia villosa* Roth) belongs to the genus *Vicia* of the tribe Vicieae (also referred to as Fabeae) [1]. It is native to Europe and West Asia and is now cultivated worldwide [2,3]. Its variant, smooth vetch (*V. villosa* Roth var. *glabrescens*), which was selected in 1926 at the Oregon station, has developed a significant differentiation due to temperature adaptation over a long period [4,5]. Hairy vetch and smooth vetch are widely cultivated as cover crops and forage, with great economic and ecological values due to their advantages in biomass production, weed suppression and nitrogen contribution [6]. Despite their widespread use, the molecular research and breeding of hairy vetch and smooth vetch progress slowly, and most investigations are limited to the screening and evaluation of germplasm resources [7,8]. As an outcrossing species with self-incompatibility, cultivated populations of hairy vetch are highly heterogeneous and usually marketed as “variety not stated” (VNS) [9,10]. Its complex and ambiguous genetic background makes it difficult to tap the full potential of this versatile species in the breeding process. Accessible genomic resources promote plant molecular breeding [11,12]. Recently, the genomes of pea (*Pisum sativum*), faba bean (*Vicia faba*), common vetch (*Vicia sativa*) and hairy vetch are now available [13,14,15,16], and the full-length transcriptome of common vetch has also been reported [17]. However, the full-length transcriptomic information of hairy vetch and smooth vetch remains unknown, while they are important for revealing the intraspecific transcriptome variation and promoting genetic improvements.

Next-generation sequencing (NGS) technology has been widely applied in transcriptome and molecular marker research, which is a high-throughput deep sequencing method with low cost [12]. Molecular markers are developed for common vetch improvement based on analysis of NGS data by comparing two subspecies [18]. In addition, RNA-Seq assemblies of two faba bean cultivars have provided valuable transcriptomes for genomics-assisted breeding [19]. PacBio single molecule real-time (SMRT) sequencing technology offers much longer read lengths and faster runs than NGS technology, making it well-suited for de novo genome and transcriptome assemblies to identify structural variation, gene isoforms and methylation [20]. By using SMRT sequencing, full-length transcriptomes have been obtained in many grass plants, such as *Tripsacum dactyloides* [21], *Carex breviculmis* [22], *Medicago falcata* [23] and *Vicia sativa* [17]. The combination of SMRT sequencing and NGS is beneficial to improve the quality of reference genome and discover novel gene loci, splicing isoforms, fusion transcripts and long non-coding RNAs (lncRNAs) [24,25].

In this study, we aimed to sequence the full-length transcriptomes of hairy vetch and smooth vetch using a combination of SMRT and NGS technologies. Based on high-quality transcripts, the functional annotations were obtained. Moreover, the prediction of coding sequence and lncRNAs, and the detection of alternative splicing events were well performed. By analysis of composite phylogeny and selection pressure, the relationship between hairy vetch and smooth vetch was well described. Our results provided valuable resources for the biological research and genetic breeding program of the vetch species.

## 2. Results

### 2.1. Phenotypic Variation of Hairy Vetch and Smooth Vetch

**Figure 1:**
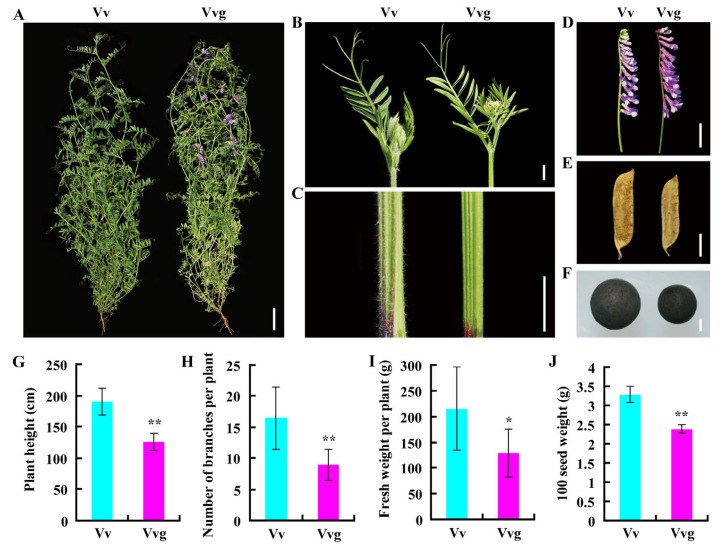
Phenotypic characteristics of hairy vetch and smooth vetch. (**A**) Plant phenotypes at heading stage in the field. Comparison of shoots apex (**B**), stems (**C**), florets (**D**), pods (**E**) and seeds (**F**) between the two vetches. Bars: 10 cm (**A**); 1 cm (**B**–**E**); 1 mm (**F**). Determination of plant height (**G**), branch number (**H**), fresh weight (**I**) and seed weight (**J**). Error bars, ±SD (*n* = 9). Student’s *t* test was used for statistical analysis (* *p* ≤ 0.05, ** *p* ≤ 0.01).

Mature plants of hairy vetch and smooth vetch showed differences in phenotype. Smooth vetch showed earlier flowering than hairy vetch (Figure 1A). Obviously, more pubescence was distributed on the surface of shoot apices and mature stems in hairy vetch than in smooth vetch (Figure 1B,C). The florets of hairy vetch and smooth vetch were violet and purple, respectively (Figure 1D). In addition, hairy vetch had larger sized pods and seeds than smooth vetch (Figure 1E,F). Plant height, primary branch number and biomass were higher in hairy vetch than in smooth vetch (Figure 1G–J).

### 2.2. Generation of Full-Length Transcriptomes Using SMRT Sequencing

To obtain comprehensive full-length transcriptomes of hairy vetch and smooth vetch, high-quality total RNAs isolated from pooled individual tissues were mixed to construct a cDNA library with a preferential size of 1 to 10 kb. A total of 37,062,620 subreads representing more than 58.67 G bases were obtained in hairy vetch, with a mean length of 1582 bp and an N50 (the scaffold length of 50% of the assembled sequences) of 2088 bp. In smooth vetch, 57,971,978 subreads representing 92.00 G bases were obtained, with a mean length of 1586 bp and an N50 length of 2036 bp (Table 1). More subreads in 0 to 4 kb region were observed in smooth vetch than in hairy vetch. In addition, a total of 764,199 and 1,192,778 reads of inserts (ROIs) were respectively generated from subreads of hairy vetch and smooth vetch after stringent filtering, with high read quality of insert (>98%) and an average depth of 42 passes (Table 1; Figure S1A,B). The distribution of ROI length was consistent with the expected cDNA size in the libraries (Figure S1C). Based on the presence of 5’ primer, 3’ primer and poly-A tail, at least 82% of ROIs were classified as full-length reads, including 608,379 and 959,314 full-length non-chimeric (FLNC) reads, with an average length of 1975 bp and 1899 bp in hairy vetch and smooth vetch, respectively (Table 1, Figure S2A). ICE algorithm clustering of FLNC reads produced 55,984 and 81,298 consensus isoforms in hairy vetch and smooth vetch; most of them (>99%) were polished, high-quality isoforms (HQs) via non-full-length sequence correction. The density plot of the length of consensus isoforms showed a peak close to 2500 bp (Figure S2B).

### 2.3. De Novo Transcriptome Assembly Using Illumina Sequencing

**Figure 2:**
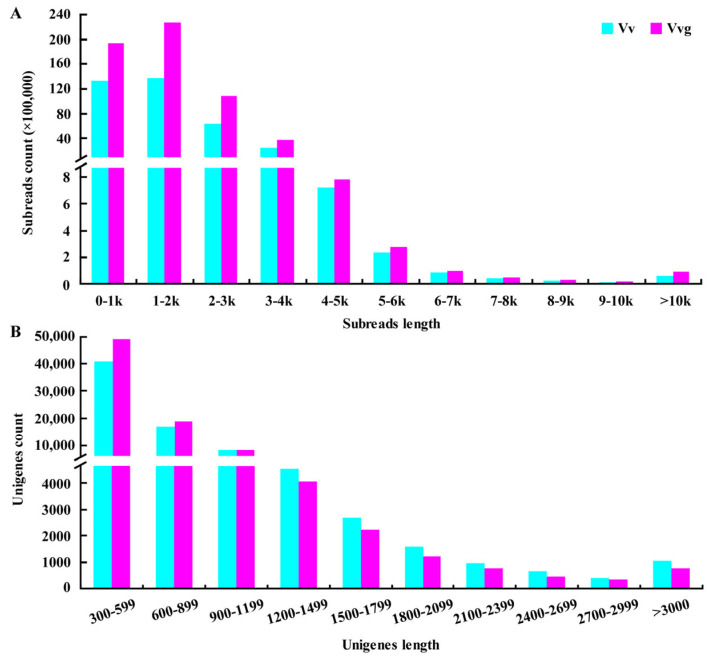
The transcripts number and length distributions of hairy vetch and smooth vetch. (**A**) The number and length distributions of subreads from SMRT sequencing. (**B**) The number and length distributions of unigenes from Illumina sequencing.

To provide a comparative reference for the PacBio isoform sequences, deep RNA sequencing of hairy vetch and smooth vetch was conducted using the Illumina NovaSeq 6000 platform, which yielded more than 20 gigabases (Gb) of clean data with high Q30 (>92%) in both cDNA libraries (Table S1). After Trinity-assembly and individual clustering, 77,755 and 85,989 unigenes were generated from 68,786,505 and 69,865,640 clean reads in hairy vetch and smooth vetch, respectively (Table 1). More than half of unigenes ranged from 300 to 599 bp in length (52.62% in hairy vetch and 57.23% in smooth vetch) and about a quarter of unigenes ranged from 600 to 899 bp (21.42% in hairy vetch and 21.68% in smooth vetch) (Figure 2). Using available NGS short reads, 322 and 524 polished low-quality isoforms (LQs) from SMRT sequencing were further corrected in hairy vetch and smooth vetch. Ultimately, any redundancy within the HQs and corrected LQs was removed, generating 28,747 and 40,600 non-redundant transcripts in hairy vetch and smooth vetch, with an average length of 1808 and 1768 bp (Table 1).

**Figure 3:**
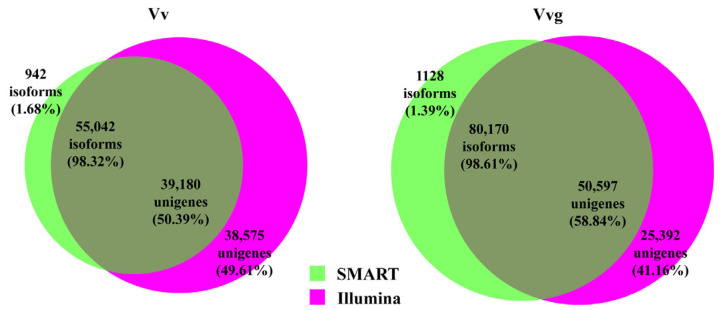
Comparison of the transcripts from SMART and Illumina sequencing in hairy vetch and smooth vetch. Consensus isoforms were from SMART sequencing and unigenes were from Illumina sequencing. The number and proportion of shared and unique transcripts were shown in the Venn diagram.

Reliable data on unigenes and consensus isoforms were compared to further understand the transcript properties. The length of unigenes ranged from 301 to 18,689 bp in hairy vetch and 301 to 20,829 bp in smooth vetch, respectively, with a mean read length and N50 less than 1000 bp and GC% < 40% (Table S2). The length of consensus isoforms ranged from 52 to 15,267 bp in hairy vetch and 53 to 11,362 bp in smooth vetch, respectively, with an average read length exceeding 1800 bp, N50 > 2200 bp and GC% > 40% (Table S2). Furthermore, 58.84% (50,597) of unigenes exhibited similarity to 98.61% (80,170) of consensus isoforms in hairy vetch using BLASTN (e-value ≤ 1 × 10^−20^, pairwise identity ≥ 75%, min bit score ≥ 100), while 50.39% (39,180) of unigenes matched 98.32% (55,042) of consensus isoforms in smooth vetch (Figure 3). Other transcripts were unique to each of the datasets.

### 2.4. Functional Annotation of the Full-Length Transcriptomes

**Figure 4:**
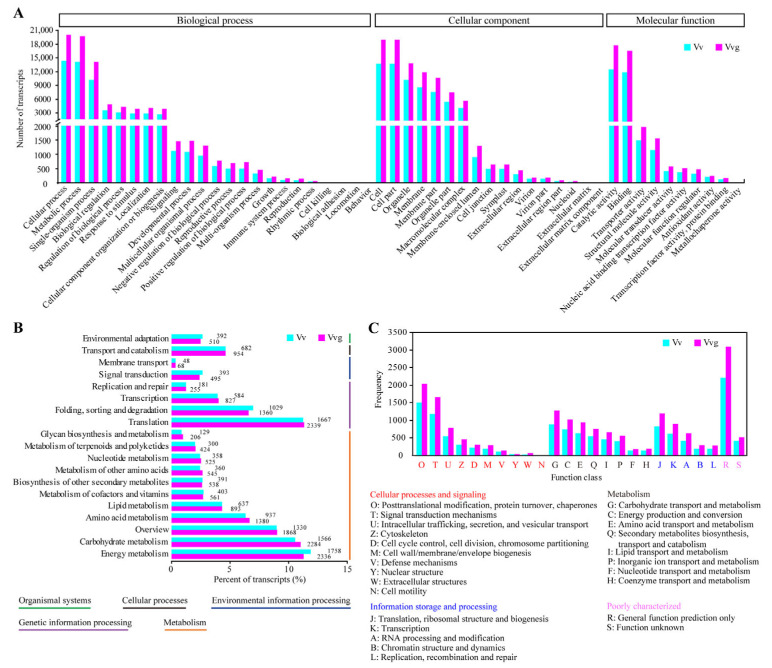
Functional classifications of the annotated transcripts from hairy vetch and smooth vetch. (**A**) The distribution of GO terms in three categories of biological process, cellular component, and molecular function. (**B**) KEGG pathway classification in five categories of organismal systems, cellular processes, environmental information processing, genetic information processing, and metabolism. (**C**) KOG functional classification in four categories of cellular processes and signaling, metabolism, information storage and processing, and poorly characterized. Color code indicates the type of categories.

Annotation of transcripts provides new insights into the biological characteristics and systematic phylogeny of the two vetches. All non-redundant transcripts of hairy vetch and smooth vetch were aligned with GO, KEGG, KOG, NR, NT and Swiss-Prot databases, respectively. As a result, 27,968 (97.29% in hairy vetch) and 39,282 (96.75% in smooth vetch) isoforms had at least one positive hit in the public databases (Table S3). A total of 7844 (27.29% in hairy vetch) and 11,014 (27.13% in smooth vetch) transcripts were overlapped in four databases (Figure S3). GO analysis showed that 22,890 and 31,952 transcripts of hairy vetch and smooth vetch were annotated in three categories of “biological processes”, “cellular component” and “molecular function”. In the “biological processes” category, the major isoforms of two vetches were assigned to “cellular component” (14,399 for hairy vetch and 20,042 for smooth vetch), “metabolic process” (14,121 for hairy vetch and 19,728 for smooth vetch), and “single-organism process” (10,188 for hairy vetch and 14,119 for smooth vetch). In the “cellular component” category, GO terms were enriched in “cell”, “cell part”, “organelle”, “membrane”, “membrane part”, “organelle part” and “macromolecular complex”. More than 50% of the GO annotated transcripts were assigned to “catalytic activity” and “binding” in the “molecular function” category (Figure 4A).

The KEGG analysis showed that all transcripts of hairy vetch (14,809) and smooth vetch (20,687) were clustered into five categories, mainly assigned to “metabolism” and “genetic information processing” pathways (Table S3; Figure 4B). In these two pathways, more than 10% of the transcripts were classified into “energy metabolism” (1758 for hairy vetch and 2336 for smooth vetch), “carbohydrate metabolism” (1566 for hairy vetch and 2284 for smooth vetch) and “translation” (1667 for hairy vetch and 2339 for smooth vetch). Using the KOG database, 11,689 and 16,421 transcripts of hairy vetch and smooth vetch were classified into 25 groups, while 1508 (12.90%), 1176 (10.06%) and 887 (7.59%) transcripts of hairy vetch arranged in the top three large groups with well-characterized function: “posttranslational modification, protein turnover, chaperones”, “signal transduction mechanisms” and “carbohydrate transport and metabolism”, along with 2043 (12.44%), 1652 (10.06%) and 1275 (7.76%) transcripts of smooth vetch (Figure 4C). In addition, based on the homology search against the NR database, over half of transcripts from hairy vetch and smooth vetch were mapped to *Medicago truncatula*, while 27.15% and 28.12% of transcripts in hairy vetch and smooth vetch were mapped to *Cicer arietinum*, respectively (Figure S4).

### 2.5. ORFs Prediction and Orthology Analysis

**Figure 5:**
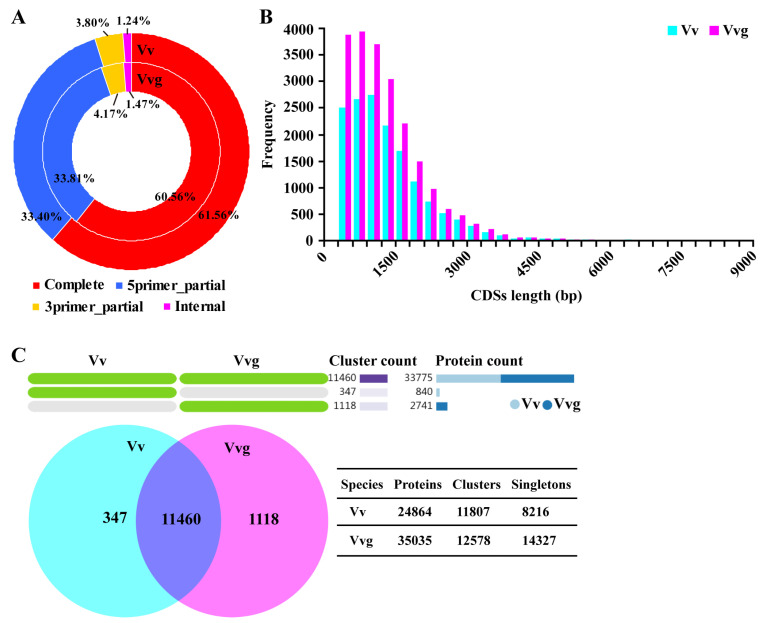
Comparison of open reading frames (ORFs) from hairy vetch and smooth vetch. (**A**) The proportions of four CDS types in two vetch transcriptomes. (**B**) The number and length distributions of complete CDSs. (**C**) Orthology analysis of two vetches by OrthoVenn2. The numbers of shared and unique clusters, singletons are shown.

A total of 24,864 and 35,035 putative ORFs encoding proteins were predicted to have a mean length of 1327 and 1279 bp in hairy vetch and smooth vetch. The complete coding sequences (CDSs) with start and stop codon accounted for more than 60% of all transcripts in the two vetches (Figure 5A). The length distributions of CDSs showed that the largest frequency ranged from 900 to 1200 bp in hairy vetch (2741 CDSs) and from 600 to 900 bp in smooth vetch (3938 CDSs) (Figure 5B). Using OrthoVenn2 web platform, orthologous cluster analysis of their protein sequences showed that 11,460 clusters were shared in both transcriptomes. The overlapped clusters were composed of 15,808 and 17,967 proteins in hairy vetch and smooth vetch, respectively (Figure 5C). There were 840 proteins corresponding to 347 clusters only in hairy vetch, and 2741 proteins corresponding to 1118 clusters only in smooth vetch. In addition, 8216 (33.04%) and 14,327 (40.89%) singletons without orthologs were presented in hairy vetch and smooth vetch (Figure 5C).

### 2.6. Alternative Splicing (AS) Detection in the Transcriptome

**Figure 6:**
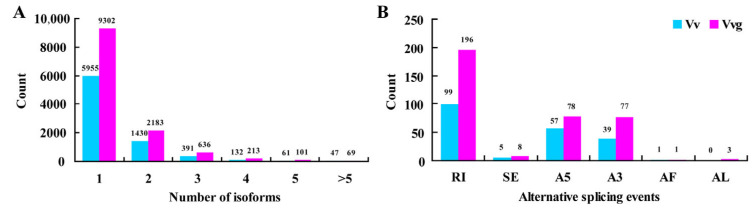
Detection and analysis of AS events in hairy vetch and smooth vetch. (**A**) Distribution of isoform numbers for UniTransModels. (**B**) Number of different types of AS events. RI, retained intron; SE, skipping exon; A5, alternative 5′ splice-site; A3, alternative 3′ splice-site; AF, alternative first exon; AL, alternative last exon.

Through systematic analysis without a reference genome sequence, the high-quality non-redundant transcripts reconstructed 8016 and 12,504 full-length UniTransModels in hairy vetch and smooth vetch (Table S4). There were similar percentages of UniTransModels, including more than one isoform in hairy vetch (25.71%) and smooth vetch (25.61%) (Figure 6A). Then, a total of 201 AS events were identified in hairy vetch, while 363 AS events were identified in smooth vetch (Table S4). The AS events mainly consisted of six types: skipping exon (SE), retained intron (RI), alternative 5′ splice site (A5), alternative 3′ splice site (A3), alternative first exon (AF) and alternative last exon (AL), while 49.25% (99) and 53.99% (196) of them belonged to retained intron (RI) in hairy vetch and smooth vetch, respectively (Figure 6B).

### 2.7. Identification of Long Non-Coding RNAs

**Figure 7:**
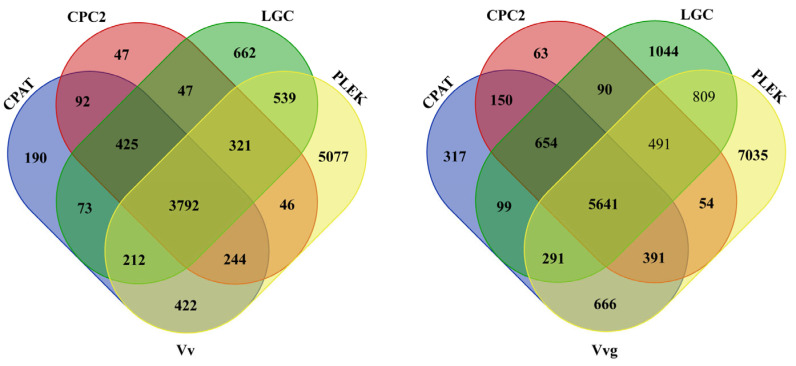
The number of predicted lncRNAs in hairy vetch and smooth vetch via CPAT, CPC2, LGC and PLEK analysis.

In addition to protein-coding RNAs, long non-coding RNA (lncRNA) is another important component of the transcriptome. There were 3792 and 5641 putative lncRNA candidates (>200 bp) predicted in hairy vetch and smooth vetch by using four methods of CPAT, CPC2, LGC and PLEK (Figure 7). After filtering out the candidates annotated by NR database, coding proteins (their length > 120 aa) and those predicted as other non-coding RNAs (tRNA, rRNA and snoRNA), 987 and 1587 high-confidence lncRNAs were obtained in hairy vetch and smooth vetch, with an average length of 604 bp (ranging from 201 to 6578 bp) and 754 bp (ranging from 201 to 5118 bp) (Supplementary Datas S1 and S2). By the homologous searching in NONCODE database, there were 257 and 327 high-confidence lncRNAs of hairy vetch and smooth vetch that correspondeded to previously discovered lncRNAs in plants, with a large proportion being newly identified lncRNAs in hairy vetch and smooth vetch.

### 2.8. Analysis of Composite Phylogeny

**Figure 8:**
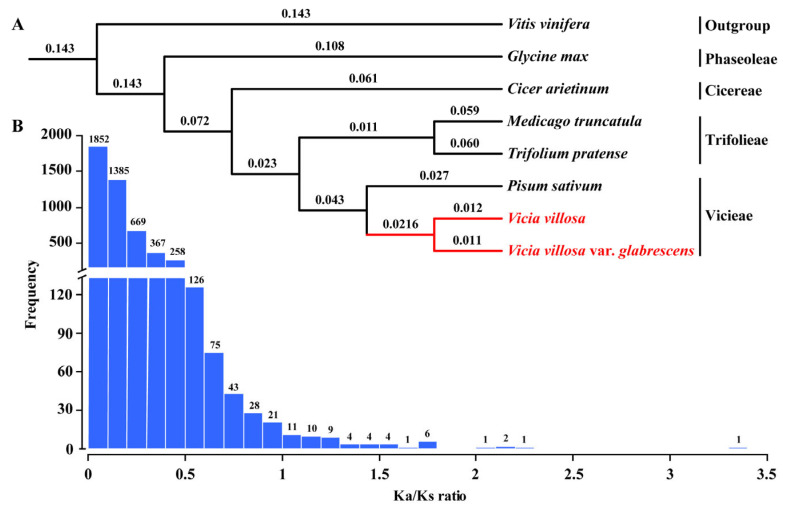
Analysis of phylogeny and selection pressure of hairy vetch and smooth vetch. (**A**) Phylogenetic relationships of hairy vetch and smooth vetch. Lineages in red utilize the *Vicia* clade. Branch lengths were labeled above the line. (**B**) Distribution of Ka/Ks ratios of orthologs between hairy vetch and smooth vetch. Ka/Ks ratios <0.5 present purifying selection, while Ka/Ks ratios >1 present positive selection.

Based on the full-length transcriptomes of two vetches, protein sequences were compared with those in five genome-sequenced legumes, including pea (*Pisum sativum*), barrel medic (*Medicago truncatula*), red clover (*Trifolium pretense*), chickpea (*Cicer arietinum*) and soybean (*Glycine max*), and a non-leguminous plant grape (*Vitis vinifera*) by using OrthoFinder algorithm. The results showed that there were 11,283 orthologous gene sets shared by hairy vetch and smooth vetch, and 12,001 orthologous gene sets shared by hairy vetch and pea, while a total of 8109 orthogroups present in eight species were identified (Table S5). Then, 121 single-copy orthogroups were selected for phylogenetic analysis. The result was consistent with known phylogenetic relationships of the Fabeae (Figure 8A) [13]. The Vicieae clade included *Vicia villosa* and was close to the Trifolieae clade, which included *Medicago truncatula*. Hairy vetch and smooth vetch were also clustered in the *Vicia* branch. They are sister groups to pea in the *Pisum* branch (Figure 8A).

### 2.9. Estimation of Selection Pressure

To estimate the selection pressure between hairy vetch and smooth vetch, an initial set of 7242 orthologous gene pairs was generated after removing the transcripts with multiple possible orthologues. There were 4878 pairs with both nonsynonymous (Ka) and synonymous (Ks < 0.1) substitutions, and they were allowed to calculate Ka/Ks ratios for estimation of selection pressure (Figure 8B). The Ka/Ks values in most orthologs (4531) were smaller than 0.5 (Table S6), suggesting that most genes likely underwent purifying selection. Only 54 gene pairs showed Ka/Ks ratios > 1 and five pairs had Ka/Ks ratios > 2, indicating that those genes underwent positive selection. Among them, a putative 3-dehydroquinate dehydratase/shikimate dehydrogenase (DHD/SDH) gene involved in the shikimate pathway underwent strong positive selection (Ka/Ks ratios > 3).

## 3. Discussion

The genus *Vicia* comprises 180 to 210 annual or perennial species representing abundant morphological, ecological and genetic diversity [1]. Within this complex, hairy vetch (genome size of ~2.04 Gb) is a very polymorphic cultivated species, which displays high tolerance to biotic and abiotic stresses [15,26]. Differently, smooth vetch lacking heavy pubescence seems to be less winter hardy [5]. In our study, significant differences in plant morphology and phenology indeed exist between hairy vetch and smooth vetch, especially in the trichome density and flowering time (Figure 1). Along with the establishment of the National Experiment Network of Green Manure (NENGM), germplasm utilization and innovation of hairy vetch has attracted much attention in China [27]. The genome and transcriptome are helpful to reveal intraspecific variation in hairy vetch and smooth vetch. Currently, insufficient genomic resources make their breeding work progress slowly.

Hybrid sequencing has become popular for studying plant transcriptomes to provide high-quality and increasingly complete assemblies [25,28]. To obtain a reliable full-length transcriptome, the multi-tissue pooled RNA libraries of hairy vetch and smooth vetch were sequenced using SMRT and NGS technologies, which generated 55,984 and 81,298 consensus isoforms corresponding to 77,755 and 85,989 unigenes from the two vetches. Although the transcripts number is lower than that of unigenes, consensus isoforms exhibit a longer mean read length (>1800 bp) and N50 (>2200 bp) (Table S2), implying higher sequence integrity for the species. Similar results were observed in a previous study of *Medicago sativa* [29]. Moreover, almost all consensus isoforms could match to unigenes (Figure 3). Compared with Illumina sequencing, the higher read accuracy (>98%) of the Pacbio Sequel II System was shown in this study. After correcting low-quality isoforms with NGS short reads, a total of 28,747 and 40,600 high-quality non-redundant transcripts were identified in hairy vetch and smooth vetch, respectively (Table 1), with close to 30,427 full-length transcripts identified in common vetch [17]. The related species, pea and faba, contain 44,785 and 34,221 genes in their genomes [13,16]. Therefore, the full-length transcriptomes of hairy vetch and smooth vetch provide relatively comprehensive reference genomic resources. Moreover, smooth vetch contains more transcripts and orthologous clusters than hairy vetch (Figure 5), revealing intraspecific variation in the transcriptome of *Vicia villosa*.

Progress in genomic sequencing has led to a rapid enrichment of protein databases. The rational annotation and classification of proteins specifying the core biological functions is critical for maximizing the use of genomic information [30]. In our study, the great majority of isoforms (more than 96%) from hairy vetch and smooth vetch were assigned in at least one protein database, while the overlapped transcripts made up less than 30%. According to the results of GO, KEGG and KOG analyses, the enriched subcategories were similar in either hairy vetch or smooth vetch due to their genetic closeness (Figure 4). Among them, “metabolic process”, particularly “carbohydrate metabolism”, is of great concern. In line with the above results, a large fraction of the genes from hairy vetch and smooth vetch were aligned and clustered into various orthologous groups (Figure 5C). Besides, NR annotation revealed that more transcripts of hairy vetch and smooth vetch matched to *Medicago truncatula* than the other Leguminous species, which might be attributed to the incompleteness of known databases of *Pisum sativum* and *Vicia faba*.

A complete transcript resource is fundamental to discovering gene isoforms and lncRNAs, especially in species without reference genomes [22,23]. AS indicates the process in which alternative splice sites are selected by the spliceosome to generate more than one transcript, is a crucial post-transcriptional regulatory mechanism in plants [31]. In diverse biological responses, AS significantly increases the complexity and flexibility of the transcriptome and proteome [32]. Based on our high-quality full-length transcriptomes, thousands of AS isoforms were identified using UniTransModels as a reference (Figure 6), which are different to those identified with genome sequence [33]. A similar AS frequency (around 25%) was detected in hairy vetch and smooth vetch, but it was lower than that in strawberry (66.43%) and cassava (77.6%) owned reference genomes [24,33], indicating that other AS events may be ignored in the two vetches. As reported, retained intron was the predominant type of alternative splicing [24,34]. The same result was shown in this study. Further accurate identification and characterization of the different AS types at a genome-wide level will shed light on post-transcriptional regulation in hairy vetch and smooth vetch.

As a novel kind of non-coding RNA longer than 200 nucleotides, lncRNAs play vital roles as gene regulators in plant development and environmental adaptation [35,36]. However, no lncRNAs have previously been reported in hairy vetch and smooth vetch. As usual, the combined forecasting method has been used to obtain the most likely candidates of lncRNAs [22,29]. Compared with the coding potential calculator CPC1, CPC2 has an advantage of fast and accurate assessment [37]. For a novel algorithm LGC, it is robustly effective in discriminating lncRNAs from protein-coding RNAs across diverse species [25]. By four analysis methods, our study predicted 3792 and 5641 putative lncRNAs for hairy vetch and smooth vetch (Figure 7). In the relative species, 7191 lncRNAs of *Pisum sativum* [13] and 2258 lncRNAs of *Medicago truncatula* (data from NONCODE database) were presented. Furthermore, 987 and 1587 high-confidence lncRNAs with an average length of 604 and 754 bp were identified in two vetches, respectively, which were shorter than the protein-coding RNAs as reported in previous research [22].

Known phylogenetic relationships across legumes with significant genome resources have been shown in their evolution [13], revealing the diversity resulting from genome size expansions and contractions in different lineages of Fabeae [38]. In addition to using the genomic data, full-length transcriptome resources were also suitable for comparative genetic and evolutionary analyses of non-model species [21,39]. It was thought that smooth vetch was derived from hairy vetch, and that they belonged to two different varieties or subspecies of the same species [5]. As expected, hairy vetch was closest to smooth vetch within a phylogenetic context (Figure 8A), consistent with the result of DNA barcoding evaluation using molecular markers [40]. Moreover, the vetches in genus *Vicia* were closely related to pea in genus *Pisum*, which provided supporting evidence for previous research based on the comparative analyses of repetitive DNAs [38].

With prevalent pod dehiscence and seed dormancy, hairy vetch does not exhibit a typical domestication syndrome [8]. As a semi-domesticated species, accessions or ecotypes of hairy vetch are predisposed to natural selection and genetic drift when seed is increased [7]. Ka/Ks values are widely used to distinguish protein-coding genes under purifying or positive selection [41]. Purifying selection was the predominant force affecting evolutionary history [42]. In line with previous studies, 4531 of 4878 orthologous pairs between hairy vetch and smooth vetch had a Ka/Ks value < 0.5 (Figure 8B), suggesting that most genes had likely undergone purifying selection, while orthologs under positive selection contained interesting candidate genes, the highest ratio of which consisted of a *DHD/SDH* gene involved in the shikimate pathway. In plants, the DHD/SDH-mediated pathway provides aromatic amino acids that are used to generate numerous secondary metabolites (i.e., pigments, flavonoids, auxins, phytoalexins, lignin and tannins) [43]. RNAi-mediated suppression of *NtDHD/SDH1* gene severely inhibited the plant height and shoot fresh weight of transgenic tobacco, which is due to the reduced content of aromatic amino acids and downstream products, such as cholorogenic acid and lignin [44]. In future work, further verification of the function of the DHD/SDH-mediated pathway will enhance the understanding of phenotypic differences and intraspecific diversity between hairy vetch and smooth vetch.

## 4. Materials and Methods

### 4.1. Plant Materials and Sample Preparation

Healthy seeds of hairy vetch ‘Turkmenistan’ (an introduced species from the former Soviet Union) and smooth vetch ‘Liangshan’ (a local variety approved by the state in 1994) were from Nanjing Agriculture University (Nanjing, Jiangsu, China). Field tests for phenotype observation and determination were conducted at the Baima Teaching and Research Base of Nanjing Agriculture University. For RNA sequencing experiment, seeds were soaked in water for 3 days and then transplanted to a nutrition medium containing peat, vermiculite and pearlite (3:1:1 by volume). The seedlings were grown in a growth chamber at 25 °C/20 °C (day/night) under a 16 h photoperiod. To obtain a wide coverage of full-length transcriptome, samples of roots, stems, leaves and shoots apex from 14-d-old seedlings, shoots apex and florets at 86-d-old stage, 14-d-old seedlings after 4 °C cold treatment for 2 h and 24 h, and 14-d-old seedlings after 400 mM NaCl treatment for 2 h and 24 h were independently collected for hairy vetch and smooth vetch, respectively. All samples were immediately frozen in liquid nitrogen and stored at −80 °C. For each tissue, at least three plants were pooled in each of three biological replicates.

### 4.2. RNA Extraction and Assessment

Total RNA was extracted using a FastPure Plant Total RNA Isolation Kit (Vazyme, Cat. No. RC401, Nanjing, China). The purity, concentration and integrity of total RNA were checked using a NanoDrop 2000 spectrophotometer (Thermo Fisher, Vacaville, CA, USA) and an Agilent 2100 Bioanalyzer (Agilent Technologies, Palo Alto, CA, USA). Qualified RNA samples with optical density (OD) 260/280 above 2.0 and RNA integrity number (RIN) ≥ 7.0 were used for further experiments.

### 4.3. Library Preparation and Sequencing

Equal amounts of total RNA from each tissue sample were mixed to construct cDNA library construction. For PacBio sequencing, the high-quality mRNA enriched by Dynabeads Oligo (dT)25 (Invitrogen, Cat. No. 61002, Carlsbad, CA, USA) was reverse-transcribed into cDNA using a SMARTer^™^ PCR cDNA Synthesis Kit (Clontech, Cat. No. 634926, Shiga, Japan). Then, the cDNA fragments (1–10 kb) after PCR amplification were selected by the BluePippin^™^ Size Selection System (Sage Science, Beverly, MA, USA). The screened cDNA was further amplified by PCR, end repaired and connected to the SMRT dumbbell-type connector. Finally, the library was generated by a secondary screening with BluePippin^™^. After the quantification and qualification of the library via Qubit 2.0 (Thermo Fisher, CA, USA) and Agilent 2100, SMRT sequencing was performed on the PacBio Sequel II platform (Pacifc Bioscience, Menlo Park, CA, USA) with one SMRT cell by Berry Genomics Biotechnology Co., Ltd. (Beijing, China).

For Illumina sequencing, total RNA from each pool was purified with Dynabeads Oligo (dT) 25 and randomly interrupted by the addition of fragmentation buffer. Using mRNA as a template, first-strand cDNA was synthesized with random hexamers, and second-strand cDNA was synthesized after the addition of DNA polymerase Ι, RNase H, dNTPs and buffer. Subsequently, the double-stranded cDNA was purified with QIAquick PCR Purification Kit (QIAGEN, Cat. No. 28106, Hilden, Germany). The purified cDNA was then subjected to end repair, the addition of a poly-A tail and ligation with sequencing linkers. The fragment size was selected via electrophoresis. Ultimately, the library was prepared by PCR-based enrichment. Based on the expected quantification and insert size, the library was applied to next-generation sequencing (NGS) on the Illumina NovaSeq 6000 platform (Illumina, San Diego, CA, USA) to generate 150 base pair (bp) sequence reads.

### 4.4. Quality Filtering and Transcriptome Assembly

The PacBio-sequencing raw reads were preprocessed with default parameters to obtain subreads (read length ≥ 50 bp and read accuracy ≥ 0.75) using the SMRT Link v8.0 Analysis software (https://www.pacb.com/, accessed on November 2019). After filtering the subreads with the requirement of full passes ≥ 0 and consensus accuracy ≥ 0.75, the reads of insert (ROIs) were generated, which could be recognized as either full-length reads or non-full-length reads by the presence of 5′ primer, 3′ primer and the poly-A tail. The full-length reads without any adapter sequence inside were classified as full-length non-chimeric (FLNC) reads. Consensus isoforms were identified using the algorithm of iterative clustering for error correction (ICE) and further polished to obtain high-quality isoforms (HQs) combining with non-full-length reads.

The Illumina-sequencing raw reads were filtered by removing adaptor sequences, ambiguous reads (‘N’ removal ratio greater than 10%) and low-quality reads (the number of bases with Q value ≤ 3 accounted for more than 50%). Subsequently, clean reads were estimated with the parameters of Q30 and GC content, and then were assembled with Trinity Release v2.4.0 (https://github.com/trinityrnaseq/, accessed on 6 February 2017) to generate high-quality unigenes. Meanwhile, error correction of low-quality isoforms (LQs) from PacBio data was conducted with NGS clean reads using the software proovread v2.14.1 (https://github.com/BioInf-Wuerzburg/proovread, accessed on 10 July 2014). The non-redundant transcripts (identity > 0.99) were further obtained by the program CD-HIT v4.8.1 (https://github.com/weizhongli/cdhit, accessed on 28 February 2019).

### 4.5. Functional Annotation of Transcripts

The non-redundant isoforms were annotated by the BLASTX searches against five public databases, including GO (Gene Ontology), KOG (Eukaryotic Orthologous Groups), KEGG (Kyoto Encyclopedia of Genes and Genomes), NR (NCBI non-redundant protein sequences), NT (NCBI nucleotide sequences) and Swiss-Prot (a manually annotated and reviewed protein sequence database). The cut-off e-values of the searches for GO, KEGG, NR, NT, Swiss-Prot were 1 × 10^−5^ and 1 × 10^−2^ for KOG.

### 4.6. Prediction of ORFs

TransDecoder v3.0.0 software (https://github.com/TransDecoder, accessed on 27 April 2016) was used to predict ORFs from the non-redundant transcripts. The candidate coding sequences (CDSs) encoding more than 100 amino acids were further identified by alignment to the Swiss-Prot database with a threshold e-value of 1 × 10^−5^, and confirmed by searching in the Pfam database. Based on the integrity of transcripts, the predicted CDSs could be divided into four types of complete (containing start and stop codons), 5′ prime partial, 3′ prime partial and internal.

### 4.7. Detection of Alternative Splicing Events

To detect AS events, the non-redundant transcripts were processed with Coding GENome reconstruction Tool (Cogent) v6.0.0 (https://github.com/Magdoll/Cogent, accessed on 12 December 2019). Each transcript was reconstructed into one or several unique transcript models (UniTransModels) using a de Bruijn graph method. Then the transcripts before Cogent reconstruction mapped to UniTransModels using GMAP v2017.06.20 (http://research-pub.gene.com/gmap/, accessed on 20 June 2017). Collapsed transcripts with different splicing junctions were identified as transcript isoforms of UniTransModels. Ultimately, SUPPA v2.3.1 software [45] was used to identify AS events, which included seven types of skipped exon (SE), mutually exclusive exon (MX), retained intron (RI), alternative 5′ splice site (A5), alternative 3′ splice site (A3), alternative first exon (AF), and alternative last exon (AL).

### 4.8. Identification of Long Non-Coding RNAs (lncRNAs)

To ensure the accuracy of the prediction, the non-redundant isoforms with lengths larger than 200 nt were evaluated for protein-coding potential using Coding Potential Assessment Tool (CPAT) [46], Coding Potential Calculator 2 (CPC2) [37], ORF Length and GC content (LGC) [25] and Predictor of long non-coding RNAs and messenger RNAs based on an improved k-mer scheme (PLEK) [47]. The putative non-coding RNAs further filtered out any NR-annotated transcripts and ORFs encoding protein longer than 120 amino acids (aa). The candidate transcripts were then used for a BLAST search against the Rfam database to eliminate other non-coding RNAs of tRNA, rRNA and snoRNA. Moreover, the high-confidence lncRNAs were searched for homologs in NONCODEV6plant database (http://www.noncode.org/) using ncbi-blast-2.2.26 with a threshold e-value of <1 × 10^−4^.

### 4.9. Construction of a Phylogenetic Tree

A dataset of syntenic orthologous genes was identified across two vetches and six genome-sequenced relative species by OrthoFinder v2.2.7 [48]. The reference genome sequences of pea (*Pisum sativum* v1a) and chickpea (*Cicer arietinum* v3) were downloaded from Pulse Crop Databases (PCD, https://www.pulsedb.org), other genome sequences of barrel medic (*Medicago truncatula* v4.0), red clover (*Trifolium pretense*), soybean (*Glycine max* v2.1) and grape (*Vitis vinifera* 12X) were available in Ensembl Plants database (http://plants.ensembl.org). Then, the gene sequences of single-copy orthogroups were aligned by MAFFT v7.474 [49]. Conservative sequences were extracted and concatenated into a supergene in each species. Based on the maximum likelihood (ML) method, phylogenies of genes were inferred using RAxML-NG with GTRGAMMA model and 1000-fold bootstrap replicates.

### 4.10. Estimation of Ka/Ks Ratios

The putative orthologous gene pairs between hairy vetch and smooth vetch were preliminarily identified by OrthoVenn2 algorithm with a threshold e-value of 1 × 10^−5^ and inflation index of 1.5 (https://orthovenn2.bioinfotoolkits.net, accessed on July 2019) [50]. A transcript in one species with multiple possible orthologues in the other species was excluded. For each orthologous gene pair, the rates of nonsynonymous (Ka) and synonymous (Ks) substitution were calculated using Simple Ka/Ks Calculator of TBtools v1.081 [51] according to the Nei–Gojobori method. The candidates with Ks > 0.1 were excluded due to the possibility of being paralogs [52]. Finally, the remaining orthologs with both Ka and Ks were further used to calculate Ka/Ks ratios for the estimation of selection pressure.

## 5. Conclusions

For the first time, comprehensive full-length transcriptomes of hairy vetch and smooth vetch were separately obtained by a combination of PacBio Iso-seq and Illumina short read sequencing method, generating a total of 28,747 and 40,600 high-quality non-redundant transcripts in hairy vetch and smooth vetch. Functional annotation and predictions of ORFs, AS events, lncRNAs were performed more reliably in our study, as well as orthology analysis across plant species. Notably, transcriptome diversity was shown in two species of *Vicia villosa*. Both the vetches were clustered together and closely related to the genus *Pisum*. Furthermore, only a fraction of orthologous genes between the two vetches were under positive selection. These new findings provide a basis for the genomic research and genetic engineering breeding of hairy vetch and smooth vetch.

## Figures and Tables

**Table 1:** Statistics of the SMRT sequencing data of hairy vetch and smooth vetch.

Samples	Vv	Vvg
cDNA size	1–10 kb	1–10 kb
SMRT cells	C01	B01
Number of subreads	37,062,620	57,971,978
Subreads bases (bp)	58,665,356,486	92,000,968,338
Mean length of subreads (bp)	1582	1586
N50 length of subreads (bp)	2088	2036
Number of reads of inserts (ROIs)	764,199	1,192,778
Read of insert bases (bp)	1,638,414,877	2,460,511,244
Mean read length of insert (bp)	2144	2062
Mean read quality of insert (%)	98.67	98.68
Mean number of passes	42	42
Number of full-length reads	631,263	989,890
Full-length percentage (%)	82.60	82.99
Number of full-length non-chimeric reads	610,398	963,051
Mean length of full-length non-chimeric reads (bp)	1975	1899
Number of full-length non-chimeric with polyA reads	608,379	959,314
Number of consensus isoforms	55,984	81,298
Number of polished high-quality isoforms	55,662	80,774
Number of polished low-quality isoforms	322	524
Percent of polished high-quality isoforms (%)	99.42	99.36
Number of non-redundant high-quality transcripts	28,747	40,600

## Data Availability

The SMRT reads and the Illumina reads generated in this study have been submitted to the BioProject database of China National Center for Bioinformation (accession number: PRJCA011776).

## Supplementary Materials

The following supporting information can be downloaded at: https://www.mdpi.com/article/10.3390/plants13233291/s1, Figure S1: Statistics of reads of inserts (ROIs) from hairy vetch and smooth vetch. Figure S2: Density plot of the sequence length in hairy vetch and smooth vetch. Figure S3: The BLAST matches of two transcriptomes from hairy vetch and smooth vetch in the KOG, NR, KEGG and Swiss-Prot databases. Figure S4: Annotation of full-length transcripts from two vetches against the NR database. Table S1: Statistics of the Illumina sequencing data of hairy vetch and smooth vetch. Table S2: Statistics of SMRT isoforms and Illumina unigenes of hairy vetch and smooth vetch. Table S3: Statistics of annotated transcripts of hairy vetch and smooth vetch. Table S4: Summary of the candidate alternative splice (AS) events. Table S5: Summary of orthogroups between relative species. Table S6: Functional annotations of 54 positive selection gene pairs between hairy vetch and smooth vetch. Supplementary Data S1: 987 high-confidence lncRNAs of hairy vetch. Supplementary Data S2: 1587 high-confidence lncRNAs of smooth vetch.
