# Supplementary material for: Full-Length Transcriptomes Reconstruction Reveals Intraspecific Diversity in Hairy Vetch (Vicia villosa Roth) and Smooth Vetch (V. villosa Roth var. glabrescens)
Source: Plants (Basel). 2024 Nov 22;13(23):3291. doi: 10.3390/plants13233291 (PMC11644882; doi:10.3390/plants13233291)
Supplement: Supplementary file 1 [file plants-13-03291-s001.zip › Supplementary Materials.pdf]

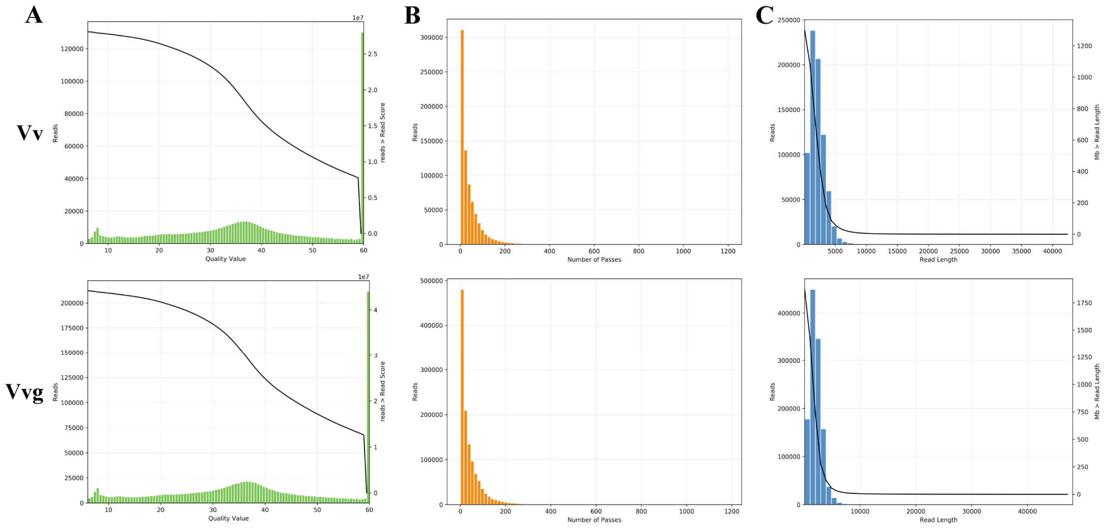

Figure S1. Statistics of reads of inserts (ROIs) from hairy vetch and smooth vetch. **(A)** Quality assessment of ROIs. **(B)** Passes number of ROIs. **(C)** Number and length distributions of reads of inserts (ROIs).

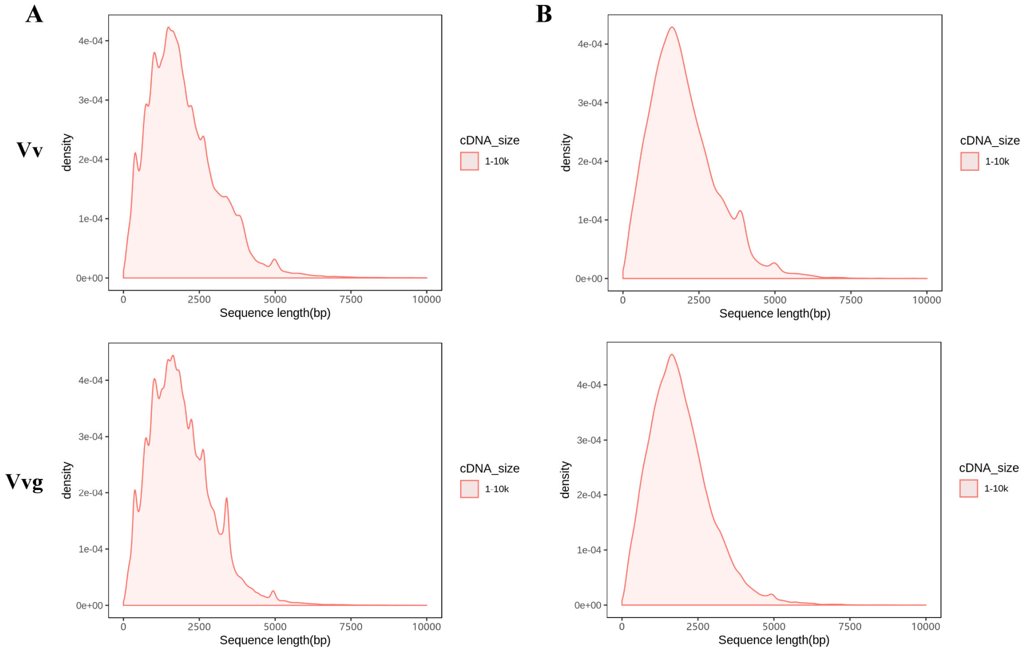

Figure S2. Density plot of the sequence length in hairy vetch and smooth vetch. **(A)** The length distributions of full-length non-chimeric FLNC reads. **(B)** The length distributions of consensus isoforms.

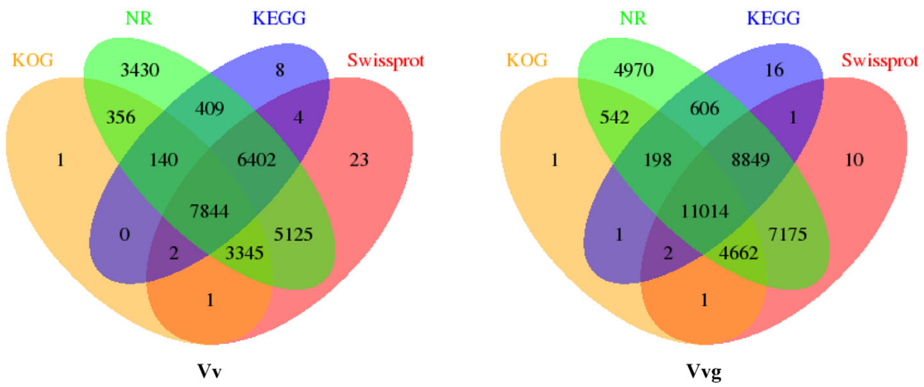

Figure S3. The BLAST matches of two transcriptomes from hairy vetch and smooth vetch in the KOG, NR, KEGG and Swiss-Prot databases.

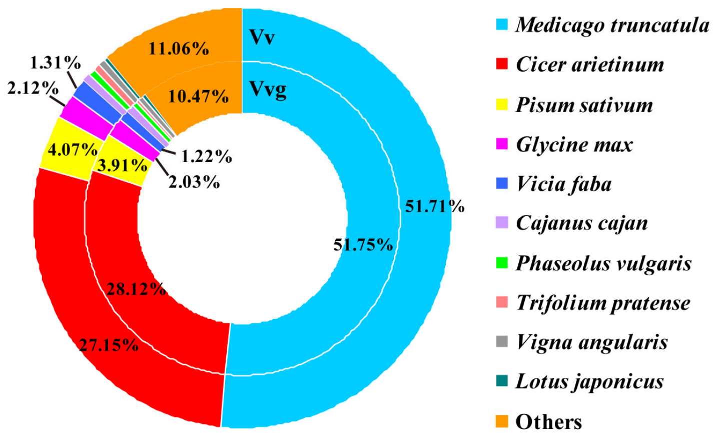

Figure S4. Annotation of full-length transcripts from two vetches against the NR database. Top-hit species were identified by homology search with the transcripts of hairy vetch and smooth vetch. The proportions of their transcripts matched homologous species were shown in the diagram.

Table S1. Statistics of the Illumina sequencing data of hairy vetch and smooth vetch.

Samples	Vv	Vvg
Number of clean reads	68,786,505	69,865,640
Clean bases (bp)	20,563,277,126	20,902,679,560
Raw Q30 (%)	93.63	92.18
Clean Q30 (%)	93.72	92.26
GC content (%)	43.45	43.51
Clean ratio (%)	99.77	99.82

Table S2. Statistics of SMRT isoforms and Illumina unigenes of hairy vetch and smooth vetch.

**Samples**	**Vv**	**Vvg**	**Vv**	**Vvg**
	Consensus isoforms		Unigenes	
Total number	55,984	81,298	77,755	85,989
Total length (bp)	112,077,981	155,681,918	60,970,488	61,308,134
Minimum length (bp)	52	53	301	301
Maximum length (bp)	15,267	11,362	18,689	20,829
Mean length (bp)	2002	1915	784	713
N50 length (bp)	2412	2270	928	802
GC content (%)	42	41	39	39

Table S3. Statistics of annotated transcripts of hairy vetch and smooth vetch.

**Database**	**Number**	**0≤length<1k**	**1k≤length<2k**	**2k≤length<3k**	**3k≤length<6k**	**length≥6k**
Vv						
GO	22,890	4506	9929	5402	2942	111
KEGG	14,809	3097	6417	3322	1887	86
KOG	11,689	1798	5311	2960	1552	68
NR	27,051	5543	11,452	6379	3532	145
NT	27,353	5981	11,366	6366	3495	145
Swiss-Prot	22,746	4132	9874	5590	3029	121
All	27,968	6288	11,550	6430	3553	147
Vvg						
GO	31,952	6486	13,878	7823	3638	127
KEGG	20,687	4434	8920	4860	2375	98
KOG	16,421	2653	7410	4357	1935	66
NR	38,016	7932	16,187	9349	4396	152
NT	38,348	8443	16,074	9307	4373	151
Swiss-Prot	31,714	5881	13,760	8137	3807	129
All	39,282	8936	16,338	9438	4418	152

Table S4. Summary of the candidate alternative splice (AS) events.

Table S5. Summary of orthogroups between relative species.

Species	Gmx	Cam	Mtr	Psa	Tpr	Vvi	Vv	Vvg
Gmx	17,854							
Cam	14,321	15,444						
Mtr	16,292	14,641	18,576					
Psa	15,852	14,522	16,396	17,856				
Tpr	16,083	14,527	16,903	16,185	18,567			
Vvi	13,823	12,566	13,534	13,436	13,585	14,595		
Vv	10,440	9904	10,450	10,582	10,482	9498	12,260	
Vvg	11,790	11,106	11,787	12,001	11,887	10,594	11,283	13,938

Abbreviation: Gmx (*Glycine max*), Cam (*Cicer arietinum*), Mtr (*Medicago truncatula*), Psa (*Pisum sativum*), Tpr (*Trifolium pratense*), Vvi (*Vitis vinifera*), Vv (*Vicia villosa*), Vvg (*Vicia villosa* Roth var. *glabrescens*).

Table S6. Functional annotations of 54 positive selection gene pairs between hairy vetch and smooth vetch.

Supplementary data S1. 987 high-confidence lncRNAs of hairy vetch

Supplementary data S2. 1587 high-confidence lncRNAs of smooth vetch
